# A randomized controlled trial of telemonitoring in older adults with multiple chronic conditions: the Tele-ERA study

**DOI:** 10.1186/1472-6963-10-255

**Published:** 2010-09-01

**Authors:** Paul Y Takahashi, Gregory J Hanson, Jennifer L Pecina, Robert J Stroebel, Rajeev Chaudhry, Nilay D Shah, James M Naessens

**Affiliations:** 1 Department of Internal Medicine Mayo Clinic 200 First Street SW Rochester, Minnesota, 55905, USA; 2 Department of Family Medicine Mayo Clinic 200 First Street SW Rochester, Minnesota, 55905, USA; 3 Division of Health Care Policy and Research Mayo Clinic 200 First Street SW Rochester, Minnesota, 55905, USA

## Abstract

**Background:**

Older adults with multiple chronic illnesses are at risk for worsening functional and medical status and hospitalization. Home telemonitoring may help slow this decline. This protocol of a randomized controlled trial was designed to help determine the impact of home telemonitoring on hospitalization. The specific aim of the study reads as follows: to determine the effectiveness of home telemonitoring compared with usual care in reducing the combined outcomes of hospitalization and emergency department visits in an at-risk population 60 years of age or older.

**Methods/Design:**

Two-hundred patients with the highest 10% Mayo Clinic Elder Risk Assessment scores will be randomly assigned to one of two interventions. Home telemonitoring involves the use of a computer device, the Intel Health Guide, which records biometric and symptom data from patients in their homes. This information is monitored by midlevel providers associated with a primary care medical practice. Under the usual care scenario, patients make appointments with their providers as problems arise and use ongoing support such as a 24-hour nurse line.

Patients will have initial evaluations of gait and quality of life using instruments such as the SF-12 Health Survey, the Kokmen Short Test of Mental Status, and the PHQ-9 health questionnaire. Patients will be followed for 1 year for primary outcomes of hospitalizations and emergency department visits. Secondary analysis will include quality of life, compliance with the device, and attitudes about telemonitoring. Sample size is based on an 80% power to detect a 36% difference between the two groups. The primary analysis will involve Cox proportional time-to-event analysis. Secondary analysis will use t-test comparisons for continuous variables and the chi square test for proportional analysis.

**Discussion:**

Patients randomized to home telemonitoring will have daily assessments of their health status using the device. Registered nurse monitoring will assess any change in status followed by videoconferencing by a mid-level provider. We obtained trial registration and Institutional Review Board approval.

**Trial registration:**

Trial registration number through http://www.clinicaltrials.gov: NCT01056640.

## Background

Older adults with multiple chronic conditions endure functional decline and loses independence. An estimated two thirds of deaths in the older population are due to chronic diseases. Five percent of patients are responsible for 50% of the costs in most healthcare systems[1]. Yet the healthcare system often places the burden of accessing healthcare on the individual patient. While this task may be easy for healthy younger adults, the older, frail population encounters numerous obstacles, including difficulty recognizing important changes in status. In response, healthcare systems have attempted to preemptively evaluate at-risk patients prior to functional decline. These methods have included more frequent prescheduled outpatient physician visits, home phone calls from the physician office, home healthcare, and home visits by physicians or midlevel providers. While each of these methods has its advantages, they are labor- and time-intensive processes that may exacerbate the healthcare worker shortage^2^. They also may miss the mark in caring for at-risk patients. Clearly, we need a more timely system of providing optimal care to chronically ill patients. Home telemonitoring, the use of audio, video, and other telecommunication technologies to monitor patient status at a distance, has emerged as a viable method for caring for this population.

Home telemonitoring may reduce hospital admissions, emergency department (ED) visits, and hospital length of stay[2]. A systematic review suggests a 20% reduction in hospitalization in home telemonitoring groups compared with the usual care in patients with cardiovascular disease[3]. However, it remains unclear if home telemonitoring will reduce hospitalizations and ED visits in older patients with multiple medical problems. We propose to answer this question in a sample of high-risk adults at least 60 years of age with mixed chronic diseases in a randomized controlled trial. Our primary aim is to evaluate the effectiveness of home telemonitoring for reducing hospitalizations, emergency department visits, and the composite outcomes of hospitalizations and ED visits compared with usual care. Our secondary aims are to evaluate the effectiveness of home telemonitoring for improving functional status, quality of life, patient attitudes, behaviors, compliance, mood, and cognitive status compared with usual care. Furthermore, we intend to evaluate the effectiveness of home telemonitoring for decreasing total healthcare costs, 30-day re-hospitalization rates, and hospital bed days.

## Methods/Design

### Study design

This multi-site, randomized controlled trial of home telemonitoring or usual care will take place in the four sites of the outpatient practice of Mayo Clinic's Division of Primary Care Internal Medicine (PCIM) in the Department of Family Medicine (FM) in Rochester, Minn. and nearby Kasson, Minn. The Mayo Elder Risk Assessment (ERA) Index will be used to randomly assign 200 high-risk patients to home telemonitoring or usual care (100 patients per intervention). Patients will be followed for 1 year after enrollment. Patients in the Rochester practice will be block-randomized as a single entity, and the patients in the Kasson practice will be block-randomized as a second entity. Subjects and providers will not be blinded to the allocation of the intervention, but analysis will be blinded to the allocation type.

### Human Subjects approval and Informed Consent

We obtained institutional review board approval from the Mayo Clinic Institutional Review Board (IRB) on Oct. 30, 2009. All patients provided written informed consent prior to enrollment and randomization. All written communication and the informed consent and protocol were reviewed by the IRB.

### Patient populations

The identification of high-risk older adults in the PCIM practice has centered on the development of the ERA, which uses the Mayo Clinic administrative databases and the Generic Disease Management System (GDMS) to electronically identify high-risk older adults who live in an assisted-living setting. Each patient older than 60 years in the PCIM and FM panel receives a score and quartile placement. The specific scoring for the ERA is detailed in Table 1. Patients with an ERA score greater than 16 will be included in the study.

**Table 1:** Scoring system of the Mayo Clinic Elder Risk Assessment

Married	-1
Age 70-79	1

Age 80-89	3

Age 90 or older	7

Race - Black	6

Race - Other	0

Race - Unknown	-6

1-5 hospital days in previous 2 years;	5

6 or more hospital days in previous 2 years	11

History of diabetes	2

History of CAD/MI/CHF	3

History of stroke	2

History of COPD	5

CAD, coronary artery disease; CHF, congestive heart failure; COPD, chronic obstructive pulmonary disease; MI, myocardial infarction.

### Exclusion criteria

Patients who live in a nursing home, have a clinical diagnosis of dementia, have a score of 29 or higher on the Kokmen Short Test of Mental Status, cannot give informed consent, or do not have the ability to use the Intel Health Guide (due to severe visual impairment and lack of a caregiver, an unwillingness to use the device, or lack of a grounded outlet or 3G mobile communications coverage) are excluded from the study.

### Demographic and practice information

Most residents of Olmsted County who are age 60 or older are female and white (>90%)[4]. While county residents are largely Northern European, the ERA index places this ethnicity as a high-risk population; thus, the potential sample may slightly over represent underserved minorities as a high-risk group.

The PCIM and FM have a combined population of about 21, 000 patients in this age group. The selection criteria of a score of 16 or greater on the ERA places these individuals in the upper decile of risk for hospitalizations, ED visits, nursing home placement, and death. The ERA is an electronically derived index number provided to all patients in the in PCIM and FM via the GDMS (Table 1).

#### Intervention - home telemonitoring

The Intel Health Guide is a U.S. Food and Drug Administration-approved device that is placed in the patient's home and connected to the health system via broadband Internet or 3G network. This device has video monitoring that allows real-time, face-to-face interaction with the provider team. Peripheral devices can be attached to measure blood pressure and pulse, oxygen saturation via pulse oximetry, glucose level via glucometer, and weight. The device incorporates a programmable, patient condition-specific touch-screen questionnaire for daily progress reporting.

The initial data collected is downloaded to the Web-based Intel Health Suite for access by the provider group via the Internet from any PC-based computer. Each patient has an individualized protocol of daily assessment questions and biometric parameters with predetermined action thresholds that are developed, revised, and monitored by the care team. An overview of the health guidelines is provided in Table 2. An example of one such parameter is the daily measurement of weight for patients with heart failure. If the weight falls outside of preset minimum or maximum values, the Intel Health Suite highlights the abnormality for the monitoring provider group.

**Table 2:** Intel Health Guide health guidelines (general)

Measurement	Normal range
Systolic blood pressure	<140 mm Hg

Diastolic blood pressure	<90 mm Hg

Pulse oximetry	>88%

Weight gain of 2 pounds/day	<2 pounds a day

Weight gain of 5 pounds/week	<5 pounds gain a week

Fasting blood sugar	80-120 mg/dL

Heart rate	60-100 beats/min.

The implementation and monitoring of the Intel Health Guide involves a team of individuals. GE Healthcare installs the system, and a clinical assistant educates the patient on how to use the Intel Health Guide and serves as a contact for questions on the device or peripherals. The protocols are determined and loaded into the device by a registered nurse (RN) and modified by a nurse practitioner (NP) overseeing the care. The patient's primary care physician oversees the overall medical care of the telemonitoring through input from the RN or NP. One RN oversees the 100 subjects on the study every day.

The system provides alerts and feedback if there is a worrisome response to a question or if vital signs are outside of preset limits. The RN communicates with the patient and seeks assistance from the subject's primary care provider or the overseeing NP for management issues. The NP or RN communicates with the patient via teleconferencing to facilitate appropriate actions, which may include limited advice, reset thresholds, timely provider outpatient visit, or ED evaluation. The use of qualified staff allows for diagnostic and treatment decisions for a wide variety of complex medical conditions that may not be amenable to protocol-based care. The participants are told to call 911 for emergencies because the Intel Health Guide is not a life-saving device.

#### Intervention - usual care

The usual care intervention includes appropriate primary care and specialty office practice visits, as required. It also includes home healthcare, post-hospital outpatient visits, a nurse-generated phone call progress report within 1 business day of hospital dismissal, and standard clinic phone triage during business hours. It also involves a 24-hour nurse triage line for questions. Patients are informed of the currently available options to patients, including the previously mentioned options, extended-hours care, and Mayo Clinic Express Care.

#### Data collection

Data will be collected by the research team and maintained electronically in the Mayo system, which is backed up daily. All paper questionnaires will be collected and entered into the electronic environment for analysis via data entry using a double-entry method. To ensure standardized processing, all investigators and study team members will be formally educated on the questionnaire and the examination instruments.

#### Data handling and security

All patient information from the Intel Health Guide will be sent to a central repository in Arizona. This information will be available to the telemonitoring clinical team. The Intel Health Guide is a secure system that complies with Health Insurance Portability and Accountability Act (HIPAA) patient safety regulations. It has a highly encrypted format in a remote database, and transmissions are secured via the communication protocol Secure Sockets Layer.

#### Assessments

All patients enrolled in the study will participate in three face-to-face visits and two phone calls. The initial visit, 6-month visit, and 12-month visit will use instruments to measure functional status, quality of life, cognition, mood, self-reported outcomes, attitudes, behaviors, and compliance. At the initial visit, the patient's Mayo current visit information and family history will be collected. At 3 months and 9 months, the study team will call the patient for descriptions of quality of life and mood. If the results indicate a risk to the patient, the patient's primary care physician will be contacted. Specifically, if the patient health questionnaire PHQ-9 indicates depression (score >14)[5], or the Kokmen Short Test of Mental Status indicates memory loss or changes of functional status (≤29)[6], this finding will be reported to the primary care physician.

The instruments used in this study measure quality of life, mood, functional status, activities of daily living, caregiver burden, and self-reported outcomes of hospitalization and ED visits. The SF-12, the short form of the SF-36, which measures quality of life and psychosocial factors[7], will determine if the quality of life has changed due to the intervention. The PHQ-9 is a validated instrument that measures depression using Diagnostic and Statistical Manual (DSM) 4 criteria for depression[5] and a scoring mechanism that can measure depressive symptoms over time. The Kokmen Short Test of Mental Status screens for cognitive decline using a 38-point screening instrument[6]. The functional measures include grip strength with tonometry, which can directly measure a person's strength in the upper extremities[8]. Both upper extremities will be tested and the best result used as the primary measure. The timed up-and-go test is a validated instrument that measures the ability to rise from a chair, turn, and return to the chair[9]. Lastly, gait speed is measured in meters per second. The patient is told to walk as quickly as possible for 6 meters. Standards for gait speed are established for older adults[10]. The Barthel Index is a self-reported questionnaire that evaluates activities of daily living for the subject[11]. The Caregiver Quality of Life Scale is used as the standard instrument for measuring caregiver stress and feelings. Outcomes include hospital admissions and visits to the ED within the past 3 months. The healthcare providers are surveyed once in the pre-stages of the study and once after completion. Providers are asked these questions:

• How confident are you in the home monitoring of your sickest patient?

• Do you feel that our practice has a good system of maintaining the functional status and health of our patients?

• How confident are you that a home-based system that allows monitoring of vital signs and symptoms will help in patient care?

• Would you be willing to recommend a home-based telemonitoring system that allows videoconferencing?

#### Outcomes measurement

The primary outcomes are hospitalizations, ED visits, and the composite outcome of hospitalizations and ED visits. Olmsted County has two primary medical centers, and all listed outcomes can be collected through the Rochester Epidemiology Project. Patient self-reports will also be used to capture care provided outside of Olmsted County, and these events will be verified by the study team. In addition, Medicare claims data will be reviewed for hospitalizations and associated costs. In practical terms, almost all patients who visit PCIM preferentially use Mayo facilities for ED and hospital care. These measures will be obtained from the medical record.

An economic analysis will be performed comparing usual care and home telemonitoring, including total non-elective care costs based on reimbursement from Medicare, Medicaid, and private insurance. Cost analysis will also use standard Centers for Medicare and Medicaid Services (CMS) costing information and reimbursement information. This will include inpatient, outpatient, and skilled nursing facility stays, and home healthcare adjusted for CMS Hierarchical Condition Codes and regional variation as well as all direct and indirect costs of the telemonitoring.

### Sample size

Using an alpha value of 0.05 and a power of 80%, the power calculations were derived from an estimated 76% event rate of hospitalizations and ED visits in 2 years in the high-risk group. Using a time-to-event approach with a 25% reduction when the sample size in each group is 352, with a total number of events required, E, of 463, a 0.050 level two-sided log-rank test for equality of survival curves will have 80% power to detect the difference between a Group 1 proportion π_1 _at time t of 0.380 and a Group 2 proportion π_2 _at time t of 0.285 (a constant hazard ratio of 0.771); this assumes no dropouts before time t. Using 100 patients in each group with a yearly hospitalization/ED rate of 38.2%, we will be able to detect a 36.1% decrease in combined outcomes. Table 3 below reflects the changes with different size groups.

**Table 3:** Sample sizes for hospitalizations and emergency department visits

Sample size	% hospitalized in intervention	% with ED/hospitalization in intervention
100	27.1 (42.5% drop)	38.2 (36.1% drop)

150	30.7 (34.8% drop)	42.1 (28.6% drop)

200	32.9 (30.1% drop)	44.4 (24.6% drop)

250	34.4 (27.0% drop)	46.0 (21.9% drop)

300	35.5 (24.6% drop)	47.2 (19.9% drop)

350	36.4 (22.7% drop)	48.1 (18.3% drop)

### Data analysis

All analysis will be performed according to group using an intention-to-treat method. The primary outcomes of combined hospitalizations and ED visits will be analyzed using a time-to-event, Cox proportional analysis. Adjustment will be made to the time-to-event analysis only if there are clear differences between the randomized groups. As a secondary method of analysis, the total number of ED visits and hospitalizations will be compared using a t-test. Initial descriptive data will be presented with means and standard deviations for parametric data. Non-parametric data will be presented with modes and 25 to 75 interquartile ranges.

Secondary data analysis will involve comparisons between the intervention group and the usual care group, with these dependent variables: SF-12 scores and sub scores, PHQ-9 scores, Kokmen Short Test of Mental Status scores, Katz Index of Independence in Activities of Daily Living scores, and Likert Scale scores for attitudes toward health and compliance (Table 4). All categorical variables will be analyzed using a t-test where appropriate, and proportional variables will be analyzed using chi square analysis for secondary outcomes. All tests for significance will use an alpha *p*-value of 0.05. Adjustments for multiple comparisons will be used for secondary analysis. All tests will assume a two-sided *p*-value.

**Table 4:** Statistical analysis for secondary outcomes

Instrument/measure	Variable type	Test	Significance
**Kokmen Short Test of Mental Status**	Continuous score	t-test	Alpha 0.05

**SF-12**	Proportional for items or continuous	Fisher's exact test or t-test	Alpha 0.05

**Likert scale scores for attitudes and behavior**	Continuous	t-test	Alpha 0.05

**PHQ-9**	Continuous	t-test	Alpha 0.05

**Katz Index of Independence in Activities of Daily Living**	Continuous	t-test	Alpha 0.05

## Discussion

Appropriate, efficient, and cost-effective care for older adults remains an important objective for both patients and providers in healthcare. It is predicted that the number of adults older than age 65 will double in the next 20 years[4]. These older adults are at higher risk for accidents and geriatric conditions such as urinary incontinence [12]. The large post-World War II cohort will require changes in how healthcare is delivered as a result of both increased numbers and required intensity of care. The utilization of efficient systems may help to mitigate some of the potential shortages of staff and resources. Telemonitoring has emerged as one possible solution to help efficiently care for older adults[13]. The potential advantages of telemonitoring are many; however, one must look at the potential costs involved with such a system.

The earliest methods of home monitoring were limited to periodic nurse telephone support, with or without other limited forms of data transmission such as cardiac rhythm monitoring. Telephone-based care has been used successfully, particularly in congestive heart failure (CHF), with a demonstrated 50% reduction in hospital readmissions through the use of nurse telephone support with remote cardiac rhythm monitoring[14]. However, the clinical desire to provide more frequent and detailed monitoring of expanded physiologic parameters and to have a remote "face-to-face" interaction with the patient has lead to the development of more sophisticated telemonitoring equipment. This equipment provides direct visual and audio communication through video monitoring and expands the ability to frequently assess patient status via capabilities (e.g., oximetry, spirometry, direct vital sign measurement, remote auscultation, blood glucose measurement). It can be monitored in a synchronous fashion to facilitate early intervention. One might easily imagine this type of technology to have the greatest impact potential for older adults who may be challenged to collect their own physiologic data and communicate well solely over the telephone.

Telemonitoring using updated equipment has been quite successful for chronic disease management. The initial use of telemonitoring targeted patients who live long distances from medical or specialty care[15]. Increasingly, telemonitoring has sought to help individuals with chronic illnesses (e.g., CHF, chronic obstructive pulmonary disease [COPD], diabetes) that require daily or frequent monitoring. The goal of telemonitoring is to identify and treat symptoms, functional decline, and other key changes in medical status before the patient requires acute care in an ED or hospital or long-term care in a skilled nursing facility. The results of many demonstration projects and pilot studies evaluating the effectiveness of telemonitoring have been favorable; however, the projects have been often limited to a single disease model. Diabetes has been widely studied in randomized controlled trials[16-18]. In the diabetic population, using a meta-analysis, on aggregate, the patients favorably endorsed home telemonitoring and had lower hospital readmissions and improved hemoglobin A1c levels[3]. In the CHF studies, all-cause mortality dropped by 40% and hospitalizations by 20%[3]. Some of the CHF studies did not demonstrate a decrease in ED visits compared with usual care[19].

Patients with mixed chronic diseases remain the most understudied group, yet many older adults have more than one chronic illness[20]. In a randomized controlled trial of 53 patients with CHF, COPD, or a chronic wound, fewer patients were re-hospitalized in the telemonitoring group, which used a device capable of videoconferencing and physiologic monitoring of vital signs, spirometry, and pulse oximetry[21]. In a similar study of 104 patients with CHF, COPD, and/or diabetes, Noel[22] demonstrated reduced bed days and ED visits when patients at home used a device capable of measuring vital signs, blood glucose levels, three-lead electrocardiography, pulse oximetry, auscultation of heart and lungs, and pain assessment. However, cost analysis was limited to a previous 6-month comparison, making interpretation difficult. Videoconferencing was not available for this study. In a recent review for the Canadian government, this study was the only publication on elderly patients with mixed chronic disease given the quality score of B (good), with the remainder deemed to have significant shortcomings[3].

The primary concern for many healthcare organizations undertaking telemonitoring is the cost justification for the clinical and capital investment. While the cost benefits of reduced utilization and functional decline are self-evident, the costs of telemonitoring have not been well-analyzed to date. In an example analysis of a single published work, the estimated annual cost for patients in the telemonitoring group was $14,678, compared with $10,161 for the usual care[3]. However, remaining methodologic issues suggested lack of comprehensive cost accounting and validated staffing protocols. Moreover, the quality of the economic evaluations to date have been deemed poor, and the report called for more studies of higher methodologic quality to include more diverse patient populations with CHF, diabetes, and COPD to increase external validity[3].

The proposed study will address the need for more rigorous study of the role of home telemonitoring in elderly patients with mixed chronic disease. We will use a unique, validated risk assessment index to systematically identify patients at high risk for hospitalization, ED visits, nursing home placement, and death. The study size will exceed the largest study to date for the mixed chronic disease population. We will provide a more robust analysis of key clinical outcomes and economic impacts not yet fully characterized and use these results in future studies comparing telemonitoring to care-transition programs. We will also use the results to define populations most suitable for these and similar interventions targeted to sustaining late-life independent living. This study may also provide a background for other monitoring devices.

The strengths of this study include the randomized trial approach, which will allow the groups to be randomly assigned and minimize the differences between the groups. The usual care group includes the standardized followup for patients, which is the standard of care for high-risk elderly patients in Rochester. The telemonitoring intervention group is based on cutting-edge technology for monitoring at home. The clinical group includes experienced midlevel nurse providers and RNs who oversee the management of home medical care. Patients also have a connection with their primary physician, who is informed of major clinical changes. Lastly, the group is maintained in a closed medical system of two major hospital groups in Olmsted County, with most care provided by the PCIM.

The major limitation is the lack of blinding inherent with two disparate groups of patients. This could lead to the Hawthorne Effect and lead to bias. It will not be practical to blind the providers or the patients receiving home equipment and constant monitoring at home. The limitations imposed will be mitigated despite the lack of blinding. Specifically, hospitalizations and ED visits as the primary outcome should occur regardless of arm or treatment and are not subjective measures. There is the potential for recall bias on self-reported outcomes; however, the primary outcomes should be captured by the medical record in nearly all cases.

## Abbreviations

SF-12: Short Form Health survey 12; PHQ-9: Patient Health Questionnaire 9; ED: Emergency Department; IRB: Institutional Review Board; PCIM: Primary Care Internal Medicine; FM: Family Medicine; ERA: Elder Risk Assessment; GDMS: Generic Disease Management System; GE: General Electric; RN: Registered nurse; NP: Nurse practitioner; HIPAA: Health Insurance Portability and Accountability Act; CMS: Center for Medicare and Medicaid Services; CHF: Congestive Heart Failure; COPD: Chronic Obstructive Pulmonary Disease

## Competing interests

The authors of the study received funding of this study through Intel and GE Healthcare through donations of the Intel Health Guide and support of the device. All authors are employed by Mayo Clinic who provided the support for this study. The authors declare no further competing interests.

## Authors' contributions

PYT and GJH were responsible for the design, implementation, data acquisition, data analysis, and reporting of the primary results. RJS and RC were responsible for data acquisition and data reporting. JLP was responsible for implementation, data acquisition, and data reporting. NDS and JMN were responsible for design and data analysis of the data, as well as data reporting. All authors have read and approve of the final manuscript.

## Pre-publication history

The pre-publication history for this paper can be accessed here:

http://www.biomedcentral.com/1472-6963/10/255/prepub
